# Red blood cell proteomics reveal remnant protein biosynthesis and folding pathways in PIEZO1-related hereditary xerocytosis

**DOI:** 10.3389/fphys.2022.960291

**Published:** 2022-12-01

**Authors:** Alexis Caulier, Nicolas Jankovsky, Emilie Fleur Gautier, Wassim El Nemer, Corinne Guitton, Hakim Ouled-Haddou, François Guillonneau, Patrick Mayeux, Virginie Salnot, Johanna Bruce, Véronique Picard, Loïc Garçon

**Affiliations:** ^1^ HEMATIM, CURS, Amiens and Laboratoire d’Hématologie, CHU Amiens, UPJV, Amiens, France; ^2^ 3P5 Proteom’IC, Institut Cochin, INSERM, CNRS, Université Paris Cité, Paris, France; ^3^ Institut Imagine-INSERM U1163, Necker Hospital, University of Paris, Paris, France; ^4^ Laboratoire d’excellence GR-Ex, Paris, France; ^5^ INSERM U1134, INTS, Paris, France; ^6^ Laboratoire d’Hématologie et Filière MCGRE, CHU Bicêtre, Le Kremlin-Bicêtre, France; ^7^ Laboratoire d’Hématologie, Faculté de Pharmacie, Université Paris Saclay, Amiens, France

**Keywords:** xeroxytosis, proteomics, piezo1 channel, ubiquitin, red blood cell

## Abstract

Hereditary xerocytosis is a dominant red cell membrane disorder characterized by an increased leak of potassium from the inside to outside the red blood cell membrane, associated with loss of water leading to red cell dehydration and chronic hemolysis. 90% of cases are related to heterozygous gain of function mutations in PIEZO1, encoding a mechanotransductor that translates a mechanical stimulus into a biological signaling. Data are still required to understand better PIEZO1-HX pathophysiology. Recent studies identified proteomics as an accurate and high-input tool to study erythroid progenitors and circulating red cell physiology. Here, we isolated red blood cells from 5 controls and 5 HX patients carrying an identified and pathogenic PIEZO1 mutation and performed a comparative deep proteomic analysis. A total of 603 proteins were identified among which 56 were differentially expressed (40 over expressed and 16 under expressed) between controls and HX with a homogenous expression profile within each group. We observed relevant modifications in the protein expression profile related to PIEZO1 mutations, identifying two main “knots”. The first contained both proteins of the chaperonin containing TCP1 complex involved in the assembly of unfolded proteins, and proteins involved in translation. The second contained proteins involved in ubiquitination. Deregulation of proteins involved in protein biosynthesis was also observed in *in vitro*-produced reticulocytes after Yoda1 exposure. Thus, our work identifies significant changes in the protein content of PIEZO1-HX erythrocytes, revealing a “PIEZO1 signature” and identifying potentially targetable pathways in this disease characterized by a heterogeneous clinical expression and contra-indication of splenectomy.

## Introduction

Hereditary xerocytosis (HX), also known as dehydrated hereditary stomatocytosis (DHST) is a dominant red cell membrane disorder characterized by an increased leak of potassium (K^+^) outside of the red blood cell (RBC), accounting for loss of water and dehydration (Gallagher 2017; Jankovsky et al., 2021). Two main genes are involved: gain of function mutations in *PIEZO1* encoding Piezo1*,* a non-selective cation channel that responds to mechanical stimuli, account for about 90% of the cases (Zarychanski et al., 2012; Albuisson et al., 2013; Andolfo et al., 2013), and gain of function mutations in *KCNN4*, encoding the Gardos channel, a Ca^2+^-dependent K^+^ channel, account for about 10% of the cases (Glogowska et al., 2015; Rapetti-Mauss et al., 2015). PIEZO1-HX presentation associates various degrees of chronic hemolysis, iron overload, pseudo hyperkalemia, perinatal edema as well as a high thrombotic risk after splenectomy (Picard et al., 2019). In most cases, hemolysis is ‘compensated’, showing increased reticulocytes and a normal hemoglobin level*.* Recently, reports pointed out dyserythropoiesis and defective reticulocyte maturation in PIEZO1-HX (Caulier et al., 2020; Moura et al., 2020), as well as defective hepcidin regulation in the liver (Andolfo et al., 2020) and increased erythrophagocytosis (Ma et al., 2021) both involved in the iron overload.

Data are still lacking about RBC and reticulocyte specificities in PIEZO1-HX. Omics have been shown to be effective approaches to identify pathophysiological mechanisms in RBC diseases. We recently published a metabolomics study in HX that identified a PIEZO1 signature characterized by an increased glycolysis rate, a decreased 2.3DPG content accounting, at least partially, for the increased hemoglobin oxygen affinity and polycythemia observed in some patients despite dyserythropoiesis and hemolysis (Kiger et al., 2021). In order to better characterize RBCs and reticulocytes in PIEZO1-HX, we have now used a proteomic approach to compare the proteome in human PIEZO1-HX and control RBCs. Indeed, we have previously developed proteomics in human reticulocytes and RBCs (Gautier et al., 2016, Gautier et al., 2018). The first proteomic studies identified more than 2000 proteins in RBCs, however, they were limited by low purity level due to 1) contamination by leucocytes or platelets and 2) poor separation between RBCs and reticulocytes, that have a significantly higher protein content, transferrin receptor, organelles, and mitochondria levels. Our improved method allowed to detect and quantify more than 2077 proteins in a highly purified population of human RBCs and reticulocytes without any contamination from leucocytes or platelets (Gautier et al., 2018). We describe here a global proteomics study of human PIEZO1-HX RBCs, aiming to identify specific patterns that may reflect PIEZO1-HX pathophysiology and represent potential therapeutic targets in this disease in which treatment are lacking since splenectomy has limited efficiency on hemolysis and leads to frequent thrombotic complications (Picard et al., 2019).

## Material and methods

Blood samples from five patients diagnosed with PIEZO1-HX (DHST 1-5) based on family history, non-spherocytic chronic hemolysis, typical osmotic gradient ektacytometry and molecular testing were obtained after informed consent according to Helsinski protocol. All patients have been already reported by our group (Picard et al., 2019) and carry four different *PIEZO1* mutations. Patients’ characteristics are summarized in Supplementary Tables S1, S2. In parallel, five EDTA blood samples from healthy donors (HD) were used as controls. Mature red cell and reticulocyte purification, protein extraction and absolute quantification were performed as described with minor differences (Gautier et al., 2018). Extensive protocols are summarized in Supplementary Material S1. Briefly, venous blood samples from HX patients or controls were first centrifuged to deplete platelets, and leukocytes and RBCs were then separated on a Ficoll gradient. Reticulocytes were labeled using Thiazole Orange staining, and cells were sorted to obtain an RBC-purified fraction (RBC-PF, Thiazole Orange negative) and a reticulocyte-purified fraction (Ret-PF, Thiazole Orange positive). Proteomic analysis was performed on the RBC-PF fraction, as reticulocyte count, although moderately increased in HX patients, was too low to enable high resolution analysis. For *in vitro-*produced reticulocytes, hematopoietic stem cells (HSC) were enriched from mobilized peripherical blood mononuclear cells (PBMCs) of four different healthy donors by CD34^+^ magnetic sorting (AutoMACS Separator, Miltenyi Biotec). CD34^+^-derived cells were cultured as previously described (Caulier et al., 2020) and exposed to DMSO or 1 µM Yoda1 from day 4 to day 24 when GPA^+^/Draq5^−^ reticulocytes were sorted on FACSAriaII. Sample preparation was performed as previously described (Gautier et al., 2018) on a Dionex U3000 RSLC nano-LC system coupled to an Orbitrap Velops mass spectrometer (Thermo Fisher Scientific, see Supplementary Material S1). Data were analyzed on a MaxQuant software (Tyanova et al., 2015) using the *Homo sapiens* Uniprot-Swissprot reviewed database. Maxquant LFQ values were imported into the Perseus software version 1.6.15.0 (Tyanova et al., 2015). Proteins expressed in at least three samples of controls or patients for erythrocytes and two samples of either condition for reticulocytes were filtered, and statistics were performed using the Student *t*-test with a *p-*value < 0.5 considered significant. The mass spectrometry proteomics data have been deposited to the ProteomeXchange Consortium *via* the Pride partner repository with the dataset identifiers PXD031963 and PXD035122 for mature RBCs and *in vitro* produced reticulocytes respectively.

## Results and discussion

**FIGURE 1:**
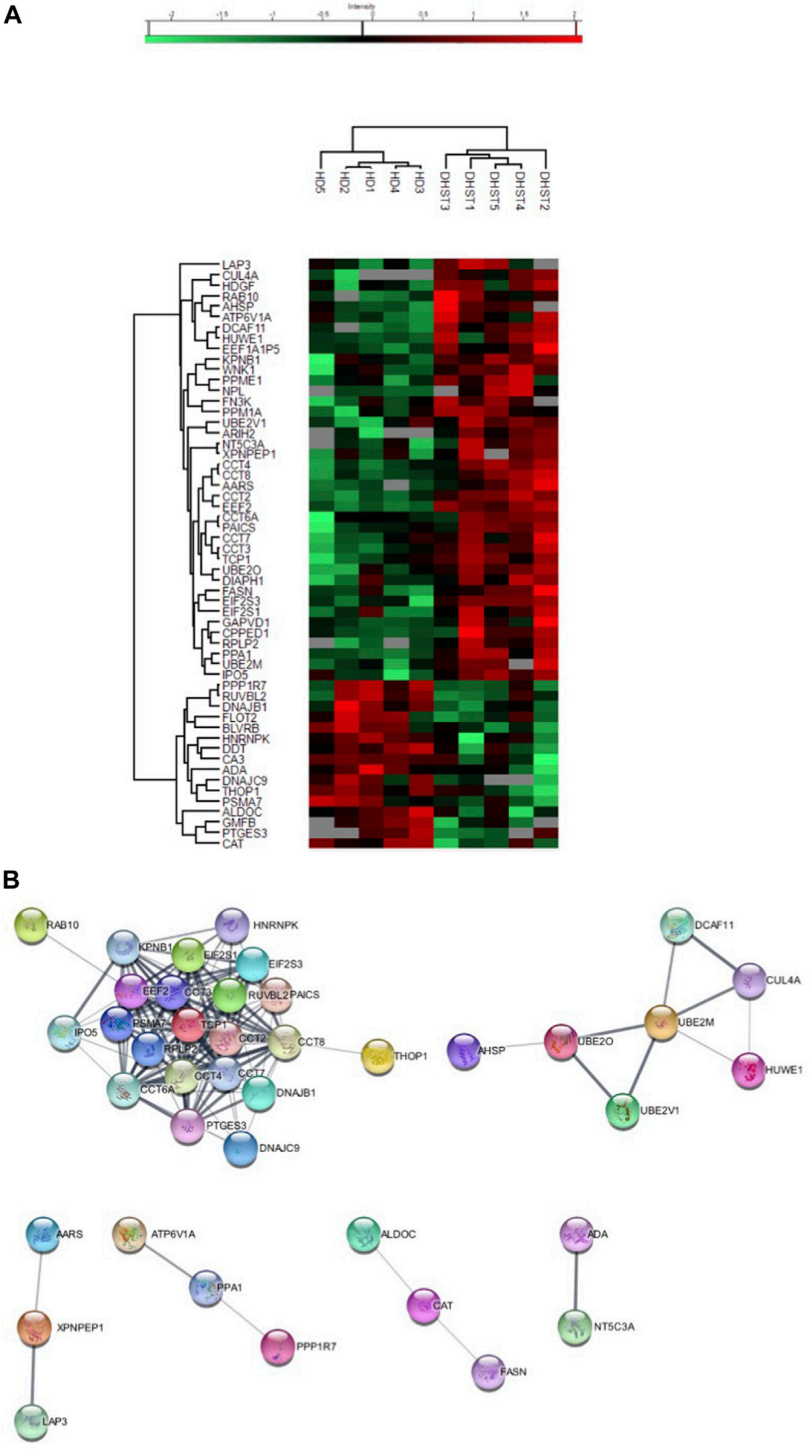
**(A)** Heatmap showing a particular PIEZO-HX (indicate as DHST) protein expression in RBCs in comparison with control (identified as HD). **(B)** Analysis working string of differentially expressed protein in PIEZO-HX (identified as DHST) in RBCs in comparison with control (identified as HD).

A total of 603 proteins were quantified in at least three samples of at least one condition (Supplementary Table S5, Supplementary Figures S1, S2). Of those, 56 were differentially expressed, 40 were overexpressed and 16 under expressed in HX RBCs compared with controls (Supplementary Table S3). The expression profile was homogenous within each group as shown by the heatmap (Figure 1A). In order to identify a PIEZO1 signature, we performed an “analysis working string” to link these differentially expressed proteins on a functional basis. As shown in Figure 1B, we could isolate a main knot containing proteins involved in protein biosynthesis and folding. The core of this knot corresponds to the Chaperonin-containing T-complex (CCT) and proteins involved in the elongation step of protein synthesis. The CCT-complex, present in all eukaryote cells, is involved in the assembly of unfolded proteins playing a role in many cellular processes such as signalling pathways and cytoskeleton assembly (Kaisari et al., 2017), regulating for example the activity of the Ca^2+^-activated form of Gelsolin (Svanström and Julie 2016). Thus, this dysregulated network observed in PIEZO1-HX *RBCs* could represent a response to the intracellular Ca^2+^-increase secondary to *PIEZO1* gain-of -function mutation. In addition to the chaperonin complex, this knot also contained proteins involved in translation and elongation such as RPLP2, EEF2, EIF2 subunit 1 and 3, all upregulated in PIEZO1-HX *RBCs*. The persistent expression of proteins involved in translation is probably reminiscent from earlier stages of erythropoiesis and may reflect the delay in erythroid precursor and reticulocyte maturation described in PIEZO1-HX (Caulier et al., 2020; Moura et al., 2020). Of note, expression of elongation factors is known to increase in response to oxidative stress as a compensatory effect (Bektaş et al., 2005; Sanchez et al., 2019). Since PIEZO1 activation increases the intracellular ROS level in nucleus pulposus (Wang et al., 2021) and activates ROS signalling in cardiomyocytes and macrophages (Geng et al., 2021; Jiang et al., 2021), expression of elongation factor that persists at a significantly high level in mature RBCs may reflect a higher sensitivity of erythroid cells carrying a PIEZO1 GOF mutation to oxidative stress. The underlying mechanisms remain unknown but could involve a decreased expression of antioxidant proteins as Catalase (CAT) or Glutathione S transferase Theta (GSTT1). If catalase was significantly down-regulated in *RBCs* from HX patients, GSTT1 protein was nearly absent in HX-*RBCs* while still expressed in controls (Supplementary Table S5). GSTT1 null polymorphism is known to induce a higher hematological toxicity after benzene exposure through an increased sensitivity to oxidative stress, and in a context of RBC disease, to worsen the severity of sickle cell disease (Nourozi et al., 2018). Among proteins absent in HX-*RBCs*, transcription elongation factor B polypeptide 1 (TCEB1) was only detected in one sample of HX-*RBCs* whereas present in four out of five controls (Supplementary Table S5), as previously described in the transcriptome of bone marrow samples from patients presenting Diamond-Blackfan Anemia (Gazda et al., 2006). This finding may participate in the dyserythropoietic features previously described in *RBCs* and reticulocytes from PIEZO1-HX patients. Of note, no retention of endoplasmic reticulum (ER) proteins has been observed in *RBCs* of PIEZO1-HX patients. It differs from observations reported in polycythemia vera (PV), where features shared with PIEZO1-HX as elevated levels of intra-cellular calcium and increased Gárdos activity, were associated with altered organelle sorting during enucleation and increased retention of ER proteins in reticulocytes and *RBCs* (Buks et al., 2021). Although sharing in part similar calcium-mediated pathways, the difference of behaviour in *RBCs* from PV and HX patients, as well as the absence of enrichment in proteins directly regulated by calcium, highlights the complexity of PIEZO1-HX, where calcium influxes fail to faithfully reveal the pathophysiology of the disease.

To further decipher the impact of PIEZO1 mutation in *RBCs*, we used a Markov Clustering (MCL) Algorithm which intends to cluster protein families more precisely (Enright, 2002) (Figure 1B). This allowed us to identify a second functional knot corresponding to proteins involved in the ubiquitination pathway such as the E2 ubiquitin-conjugating enzymes and the E3 ubiquitin-ligase HUWE. Cullin-4A (CUL4A) and DDB1- and CUL4-associated factor 11 (DCAF11) were significantly increased in PIEZO1-HX and are parts of the E3 ubiquitin ligase complex. ARIH2, another E3 ubiquitin-protein ligase that cooperates with Cullin-5 in an E3-E3 complex was overexpressed in PIEZO1-HX *RBCs* as well. We think that their persistence at higher level is the reflexion of defects occurring at earlier stages of erythropoiesis. Therefore, HX pathophysiology involves deregulation of the protein quality control pathways, particularly the Ubiquitin-Proteasome System (UPS), which was by the way first discovered in reticulocytes (Ciehanover and Hod, 1978). Several reports highlighted the crucial role of UPS in erythroid differentiation, including enucleation and reticulocyte maturation through different processes such as detoxification of alpha chains in excess, elimination of misfolded or damaged proteins, degradation of activated EPO-R or on the contrary by avoiding protein degradation by de-ubiquitining enzymes such as USP7 which stabilizes GATA1 in erythroid cells through a direct interaction at protein level (Etlinger, 1977; Chen et al., 2002; Walrafen et al., 2005; Khandros et al., 2010; Liu et al., 2010; Liang et al., 2019). Moreover, CUL4A’s downregulation is known to promote cell cycle exit and erythroid maturation by modulating key erythroid regulators such as GATA1 and P27 (Li et al., 2006). Thus, persistence of a high CUL4A expression in PIEZO1-HX, in addition to the deregulated UPS pathway could explain the delayed maturation described in PIEZO1-HX reticulocytes (Moura et al., 2020).

**FIGURE 2:**
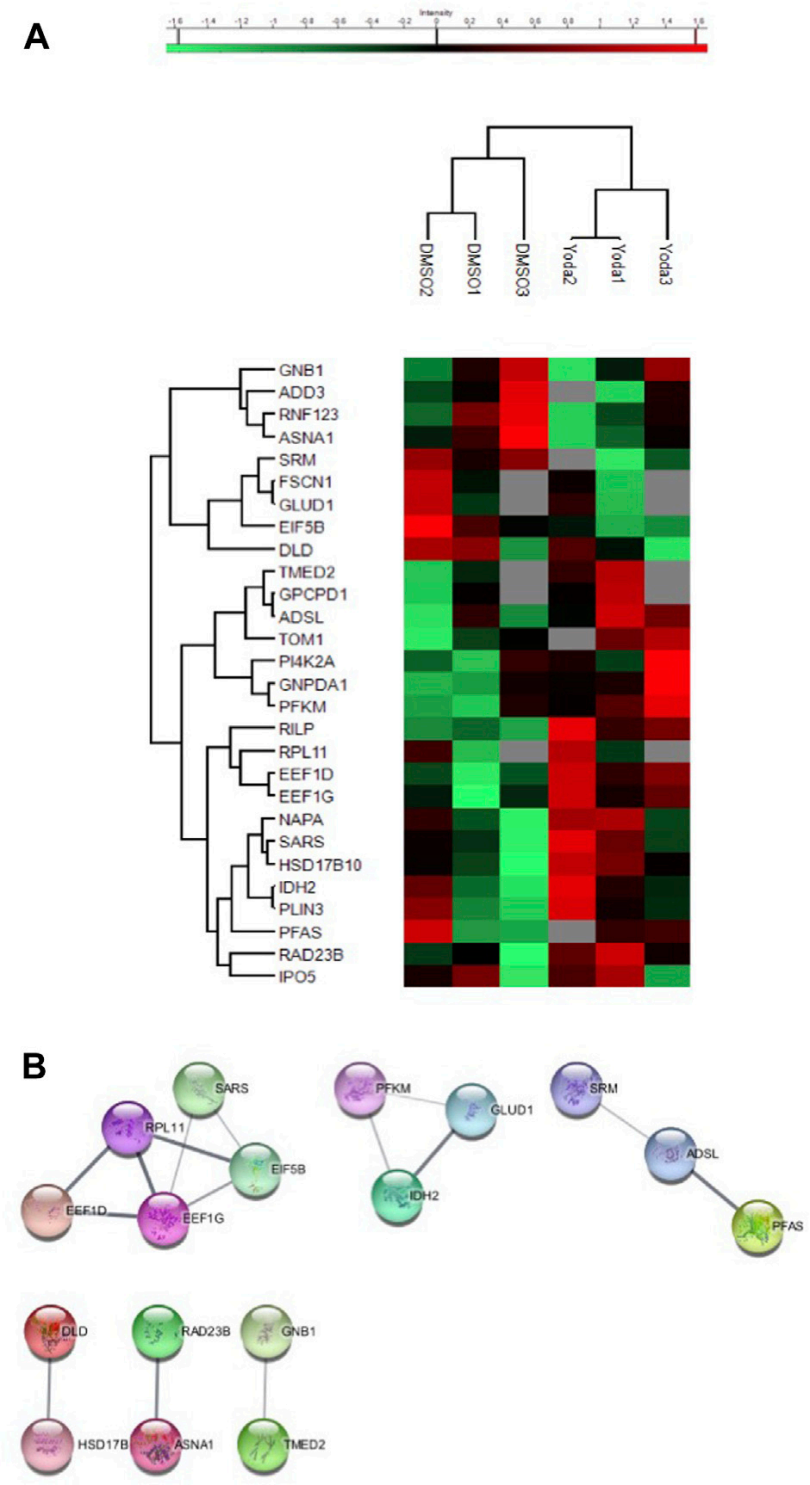
**(A)** Heatmap showing the proteins differentially expressed in CD34^+^-derived reticulocytes after DMSO or Yoda1 exposure. **(B)** Analysis working string after Markov Clustering obtained from CD34^+^-derived reticulocytes exposed to DMSO or YODA1, showing one main knot containing protein involved in protein biosynthesis.

To confirm that our findings were specifically related to *PIEZO1*-activation, we performed a proteomic analysis on reticulocytes produced *in vitro* from CD34^+^ cells either exposed to DMSO as a control or to the PIEZO1 activator Yoda1 (Syeda et al., 2015). The analysis could not be performed on mature erythrocytes because *in vitro* erythroid differentiating protocols have been shown to produce primarily reticulocytes rather than mature erythrocytes (Giarratana et al., 2011). A total of 1,169 proteins were quantified in at least two samples in at least one condition (Supplementary Table S5, Supplementary Figures S3, S4). Of those, 28 proteins were differentially expressed, 19 were overexpressed and nine under expressed in reticulocytes exposed to Yoda1 compared with controls (Figure 2A and Supplementary Table S4). Of note, expression of the transferrin receptor was similar in both conditions, ruling out the possibility that reticulocytes could be at different maturation steps. After performing a MCL Algorithm on the differentially expressed proteins, we identified functional knots of proteins involved in biosynthesis such as elongation factors EIF5B, EEF1G, EEF1D and riboprotein RPL11, confirming the results obtained with primary *RBCs* from PIEZO1-mutated HX (Figure 2B). However, we did not identify a knot of proteins involved in protein quality control pathways. This discrepancy may reflect the difference in intensity and mechanism of PIEZO1 activation induced by Yoda1 or by gain-of function mutations in HX. Furthermore, subtle changes in the proteome of reticulocytes exposed to Yoda1 reinforce our previous results, where activation of PIEZO1 mainly slowed down the differentiation of erythroid progenitors without significantly impairing the terminal maturation and enucleation steps (Caulier et al., 2020).

In summary, we present here a descriptive extensive proteomic study revealing a “PIEZO1 signature” characterized by specific modifications in RBC protein content. Our study confirms that gain of function PIEZO1 mutations, in addition to modifying erythrocyte hydration by secondary activating the Gárdos channel, affect several aspects of erythroid cell physiology, resulting in deregulation of multiple cellular processes at the protein level, with a particular focus on elongation, post-translational folding and protein quality control pathways. These data reinforce the pathophysiological knowledge of this rare disease, and offer new potential targets for the future.

## Data Availability

The mass spectrometry proteomics data have been deposited to the ProteomeXchange Consortium via the PRIDE partner repository (Perez-Riverol et al., 2022) with the dataset identifier PXD031963 (mature RBC DHS vs. HD) and PXD035122 (reticulocytes DMSO vs. Yoda1).
