# Supplementary material for: Red blood cell proteomics reveal remnant protein biosynthesis and folding pathways in PIEZO1-related hereditary xerocytosis
Source: Front Physiol. 2022 Dec 1;13:960291. doi: 10.3389/fphys.2022.960291 (PMC9751340; doi:10.3389/fphys.2022.960291)
Supplement: Supplementary file 1 [file DataSheet1.PDF]

Supplementary Methods

Quantitative comparative global Label free proteomic approach:

The proteomic approach used in the study is the quantitative label free approach (published by Cox et al., 2014). This approach allows relative and absolute quantification. Details of the analysis is described below.

Process to obtain purified erythrocytes:

Blood from HX patients and controls was centrifuged 10 minutes 150g. Plasma was isolated and centrifuged to eliminate platelets. Plasma depleted from platelets was then pooled with the red cell fraction. Cells were then separated using two different histopaque layers using Histopaque-1077 and Histopaque-1119 (Sigma-Aldrich) to remove mononuclear blood cells and granulocytes. To eliminate reticulocytes and obtain highly purified erythrocytes, nucleic acids were labelled with thiazole orange and erythrocytes were negatively sorted by FACS using a BD FACSJazz cell sorter.

Sample processing for nLC-MS/MS analysis of erythrocytes:

Cells were lyzed in lysis buffer (Tris HCl 200 mM, pH8.5, 2% SDS) and boiled 5 minutes at 95°C. Protein concentrations were determined using a bicinchoninic acid assay (BCA kit, Pierce). After reduction and alkylation in 20 mM TCEP [(tris(2-carboxyethyl)phosphine] and 50 mM Chloroacetamide, 50 µg of proteins were digested using FASP method. Eluted peptides were then fractionated using strong cation exchange (SCX) StageTips in 5 fractions as previously described (Kulak et al., 2014) and vacuum-dried while centrifuge in a Speed Vac (Eppendorf). Mass spectrometry (MS) analyses were performed on a Dionex U3000 RSLC nano-LC system coupled to an Orbitrap velos mass spectrometer (Thermo Fisher Scientific). Peptides from each SCX fraction were solubilized in 0.1% trifluoracetic acid containing 10% acetonitrile and separated on a C18 column (2-mm particle size, 75-mm inner diameter, 25-cm length; Thermo Fisher Scientific) with a 3-hour gradient starting from 99% solvent A (0.1% formic acid) and ending with 55% solvent B (80% acetonitrile, 0.085% formic acid). The mass spectrometers acquired data throughout the elution process and operated in a data-dependent scheme with full MS scans acquired with the LTQ-Orbitrap, followed by up to 20 MS/MS CID spectra in the Velos linear trap on the most abundant ions detected. Settings for Orbitrap Velos were: full MS automatic gain control (AGC): 1.10^{6}, maximum ion injection time (MIIT): 500 ms, resolution: 6.10^{4}, m/z range 400-2000 and for MS/MS, AGC: 5.10^{3}, MIIT: 200 ms, minimum signal threshold: 500, isolation width: 2 Th, peptides with undefined charge state or charge state of 1 were excluded from fragmentation. Dynamic exclusion time was set to 30 s and spectra were recorded in profile mode.

Data processing of erythrocyte data:

The mass spectrometry data were analyzed using Maxquant version 1.5.3.30(Cox et al., 2014; Cox and Mann, 2008). The database used was a concatenation of human sequences from the Uniprot-Swissprot database (release 2017-05) and the list of contaminant sequences adapted from Maxquant. The enzyme specificity was trypsin. Carbamidomethylation of cysteins was set as constant modification and acetylation of protein N-terminus and oxidation of methionines

were set as variable modification. Second peptide search was allowed and minimal length of peptides was set at 7 amino acids. False discovery rate (FDR) was kept below 1% on both peptides and proteins. Label-free protein quantification (LFQ) was done using both unique and razor peptides. At least 2 such peptides were required for LFQ ratio. The "match between runs" (MBR) option was allowed with a match time window of 1 min and an alignment time window of 30 min.

Process to obtain cultured purified reticulocytes:

CD34+ cells obtained from mobilized PBMCs of healthy donors were magnetically sorted on AutoMACS Separator (Miltenyi Biotec) using CD34+ magnetic antibodies (Miltenyi Biotec), after 30min incubation. CD34+ cells were then washed and cultured in erythroid differentiating media as described in Caulier et al., 2020. Yoda 1µM (Sigma-Aldrich) or iso-volume of DMSO was added to the media from day 4 to the end of the culture, and refreshed at each media renewal time-point. At day 24, enucleated reticulocytes were co-stained using anti-human CD235a antibody (Miltenyi Biotec) and DRAQ5 cell-permeant nucleic acid staining (Thermo Fisher Scientifics) following manufacturer's recommendation, then sorted by FACS using a BD FACSAriaII sorter.

Sample processing protocol for reticulocytes:

Cells were lyzed in lysis buffer (Tris HCl 200 mM, pH8.5, 2% SDS) and boiled 5 minutes at 95°C. Protein concentrations were determined using a bicinchoninic acid assay (BCA kit, Pierce). After reduction and alkylation in 20 mM TCEP [(tris(2-carboxyethyl)phosphine] and 50 mM Chloroacetamide, 50 µg of proteins were digested using FASP method. Eluted peptides were then fractionated using strong cation exchange (SCX) StageTips in 5 fractions as previously described (Kulak NA et al., Nat Methods, 2014) and vacuum-dried while centrifuge in a Speed Vac (Eppendorf). Mass spectrometry (MS) analyses were performed on a Dionex U3000 RSLC nano-LC system coupled to an Fusion mass spectrometer (Thermo Fisher Scientific). Peptides from each SCX fraction were solubilized in 0.1% trifluoracetic acid containing 10% acetonitrile and separated on a C18 column (2-mm particle size, 75-mm inner diameter, 25-cm length; Thermo Fisher Scientific) with a 3-hour gradient starting from 99% solvent A (0.1% formic acid) and ending with 55% solvent B (80% acetonitrile, 0.085% formic acid). MS data acquisition was performed throughout the elution process in a data-dependent scheme (top speed mode in 3 seconds) with full MS scans acquired with the orbitrap detector, followed by HCD peptide fragmentation and Ion trap fragment detection of the most abundant ions detected in the MS scan. Mass spectrometer settings for full scan MS were: 1.0E6 AGC, 60,000 target resolution, 350-1500 m/z range, maximum ion injection time (MIIT) of 60 ms. HCD MS/MS fragmentation was permitted for 2-7+ precursor ions reaching more than 5.0E3 minimum intensity. Quadrupole-filtered precursors within 1.6 m/z isolation window were fragmented with a Normalised Collision Energy setting at 30. 1.0E5 AGC Target and 60 ms MIIT were the limiting ions accumulation values. A 30 seconds dynamic exclusion time was set.

Data processing protocol for reticulocyte data:

The mass spectrometry data were analyzed using Maxquant version 2.0.3.0 (Cox et al., 2014; Cox and Mann, 2008). The database used was a concatenation of human sequences from the Uniprot-Swissprot database (release 2021-07) and the list of contaminant sequences adapted from

Maxquant. The enzyme specificity was trypsin. Carbamidomethylation of cysteins was set as constante modification and acetylation of protein N-terminus and oxidation of methionines were set as variable modification. Second peptide search was allowed and minimal length of peptides was set at 7 amino acids. False discovery rate (FDR) was kept below 1% on both peptides and proteins. Label-free protein quantification (LFQ) was done using both unique and razor peptides. At least 2 such peptides were required for LFQ ratio. The “match between runs” (MBR) option was allowed with a match time window of 1 min and an alignment time window of 30 min.

Supplementary Tables and Figures

Supplemental Tables 1 and 2

Biological, genetics (Table 1) and clinical (Table 2) characteristics of the 5 PIEZO-1HX patients. (Picard et al., 2019). When multiple mutations are found in PIEZO1 sequence, the more likely to pathogenic is highlighted in black.

	PIEZO1 mutation		GR (T/L)	Hb (g/L)	VGM (fL)	Retic (G/L)	MCHC (g/L)	MHC (pg)	Proteins/cell (µg)
**DHST1**	c.7479_7484dup	p.Glu2495_Leu2496dup	2.81	102	100	221	362	36.3	60
**DHST2**	c.1792G>C	p.Val598Leu	3.61	132	103	320		34.2	59
**DHST3**	c.6058G>A	p.Ala2020Thr	4.6	140	83.2	161	363	30.2	42.15
**DHST4**	c.2344G>A<br>c.2423G>A<br>c.1792G>A	p.Gly782Ser<br>p.Arg808Gln<br>p.Val598Met	4.2	147	99.6	225	350	34.9	66
**DHST5**	c.2344G>A<br>c.2423G>A<br>c.1792G>A	p.Gly782Ser<br>p.Arg808Gln<br>p.Val598Met	2.5	171	92.7	260	355	32.9	52
**HD1**			5.07	150	88		334	29.5	49
**HD2**									
**HD3**			4.72	134	85		336	28.4	48.8
**HD4**			4.99	141	85		239	28.2	48
**HD5**									

	Age/sexe	Hemolysis	Iron overload	Perinatal edema	Pseudohyperkaliema
**DHST1**	30/F	Y	N	Y	N
**DHST2**	47/F	Y	N	NA	N
**DHST3**	38/M	Y	Y	Y	Y
**DHST4**	38/F	Y	N	Y	Y
**DHST5**	58/F	Y	Y	N	Y

Supplemental Table 4. Differentially expressed proteins between RBCs from HX (DHST) or control (HD) patients, and their corresponding gene names.

Expression	Majority protein IDs	Gene names	Log2 Ratio DHST vs HD	Mean Log2 DHST	Mean Log2 HD
**UP**	P13639	EEF2	3.53039	23.6766	20.1462
**UP**	Q5VTE0;P68104;Q05639	EEF1A1P5;EEF1A1;EEF1A2	2.86212	22.6271	19.765
**UP**	P05387	RPLP2	2.70475	24.7741	22.0693
**UP**	P49327	FASN	1.68271	23.4464	21.7637
**UP**	P22234	PAICS	1.59141	26.7542	25.1628
**UP**	P49588	AARS	1.47477	21.7918	20.317
**UP**	P41091;Q2VIR3	EIF2S3;EIF2S3L	1.42386	23.2385	21.8146
**UP**	P61026	RAB10	1.37945	23.7263	22.3468
**UP**	Q9NZD4	AHSP	1.29019	27.1183	25.8281
**UP**	O00410	IPO5	1.23228	24.8268	23.5945
**UP**	P61081	UBE2M	1.19741	23.0832	21.8858
**UP**	Q13404	UBE2V1	1.17481	24.6161	23.4412
**UP**	Q8TEB1	DCAF11	1.15555	21.0564	19.9009
**UP**	P40227	CCT6A	1.05545	26.8018	25.7464
**UP**	P05198	EIF2S1	1.0208	22.7815	21.7607
**UP**	Q9BRF8	CPPED1	1.00322	23.6268	22.6235
**UP**	P28838	LAP3	0.975095	21.1423	20.1672
**UP**	P78371	CCT2	0.93137	27.9793	27.0479
**UP**	Q9BXD5	NPL	0.918223	21.4047	20.4864
**UP**	P49368	CCT3	0.908663	26.4288	25.5201
**UP**	P38606	ATP6V1A	0.893164	23.3632	22.47
**UP**	Q14974	KPNB1	0.889114	25.2223	24.3332
**UP**	P17987	TCP1	0.863143	26.3884	25.5253
**UP**	P50990	CCT8	0.853585	27.1768	26.3232
**UP**	P50991	CCT4	0.805005	26.6121	25.8071
**UP**	Q9NQW7	XPNPEP1	0.757181	21.7242	20.967
**UP**	O95376	ARIH2	0.755634	21.2815	20.5258
**UP**	Q9H4A3	WNK1	0.706381	23.0174	22.311
**UP**	Q7Z6Z7	HUWE1	0.699574	23.3426	22.643
**UP**	Q9C0C9	UBE2O	0.696561	25.4433	24.7467
**UP**	Q15181	PPA1	0.681112	24.6693	23.9882
**UP**	P35813	PPM1A	0.659594	22.977	22.3174
**UP**	Q9Y570	PPME1	0.594861	22.9409	22.346

**UP**	Q99832	CCT7	0.569999	26.2551	25.6851
**UP**	O60610	DIAPH1	0.539076	22.5966	22.0576
**UP**	Q9H479	FN3K	0.513196	24.1412	23.628
**UP**	Q14C86	GAPVD1	0.500277	23.7397	23.2394
**UP**	Q9H0P0	NT5C3A	0.490962	21.7565	21.2656
**UP**	Q13619	CUL4A	0.484958	20.993	20.5081
**UP**	P51858	HDGF	0.402851	21.922	21.5191
**DOWN**	P04040	CAT	-0.364452	31.4532	31.8177
**DOWN**	O14818	PSMA7	-0.384709	25.3047	25.6894
**DOWN**	Q14254	FLOT2	-0.44942	25.9827	26.4321
**DOWN**	P30043	BLVRB	-0.503589	29.4556	29.9591
**DOWN**	Q15435	PPP1R7	-0.566836	22.2903	22.8571
**DOWN**	Q9Y230	RUVBL2	-0.627031	22.587	23.214
**DOWN**	P25685	DNAJB1	-0.633029	21.4903	22.1233
**DOWN**	Q8WXX5	DNAJC9	-0.727389	20.0992	20.8266
**DOWN**	P09972	ALDOC	-0.739787	24.2868	25.0266
**DOWN**	P00813	ADA	-0.747832	19.8155	20.5634
**DOWN**	P07451	CA3	-0.793649	24.9773	25.771
**DOWN**	P52888	THOP1	-0.819527	21.4351	22.2546
**DOWN**	P61978	HNRNPK	-0.91101	22.7315	23.6426
**DOWN**	P60983	GMFB	-1.21584	21.815	23.0309
**DOWN**	Q15185	PTGES3	-1.45803	21.6497	23.1077
**DOWN**	P30046;A6NHG4	DDT;DDTL	-1.90962	23.2856	25.1952

Supplemental Table 5. Differentially expressed proteins between cultured erythrocytes from HX (DHST) or control (HD) patients, and their corresponding gene names.

Expression	Majority protein IDs	Gene names	Log2 Ratio DMSO vs Yoda1	Mean Log2 DMSO	Mean Log2 Yoda1
**UP**	P46926;P46926-2	GNPDA1	-0.181714	17.1973	17.379
**UP**	O00410;O00410-3;O00410-2	IPO5	-0.214439	18.5136	18.728
**UP**	P54727;P54727-2	RAD23B	-0.290618	19.9471	20.2377
**UP**	P08237-3;P08237;P08237-2	PFKM	-0.373775	17.3758	17.7495
**UP**	O15067	PFAS	-0.374416	17.3303	17.494
**UP**	P54920	NAPA	-0.497541	18.7686	19.2662
**UP**	P49591	SARS	-0.507633	18.3372	18.8449

**UP**	O60664-4;O60664;O60664-3	PLIN3	-0.528048	17.185	17.713
**UP**	Q9NPB8	GPCPD1	-0.545201	15.4597	16.0049
**UP**	P30566;P30566-2	ADSL	-0.581664	19.24	19.8216
**UP**	P48735;P48735-2	IDH2	-0.721919	19.7475	20.4694
**UP**	O60784;O60784-4;O60784-2;O60784-3	TOM1	-0.791096	14.9676	16.0709
**UP**	Q9BTU6	PI4K2A	-0.803885	16.4078	17.2117
**UP**	P62913-2;P62913	RPL11	-0.829947	18.1515	18.9815
**UP**	Q15363	TMED2	-0.879981	15.089	15.969
**UP**	P26641;P26641-2	EEF1G	-0.947262	19.4551	20.4024
**UP**	Q99714;Q99714-2	HSD17B10	-1.00174	17.4466	18.4484
**UP**	Q96NA2	RILP	-1.14654	17.1807	18.3272
**UP**	P29692-2;P29692;P29692-4	EEF1D	-1.5352	18.5803	20.1155
**DOWN**	Q9UEY8-2;Q9UEY8	ADD3	1.36835	16.7308	15.7695
**DOWN**	O60841	EIF5B	1.24286	18.0322	16.7894
**DOWN**	Q16658	FSCN1	0.746424	17.3295	16.5831
**DOWN**	P62873;P62873-2	GNB1	0.671961	18.4198	17.7479
**DOWN**	P00367;P00367-3;P00367-2;P49448	GLUD1;GLUD2	0.560844	17.0853	16.5245
**DOWN**	O43681	ASNA1	0.498727	19.3087	18.81
**DOWN**	P19623	SRM	0.429128	16.8525	16.3819
**DOWN**	Q5XPI4;Q5XPI4-2	RNF123	0.361284	17.9386	17.5773
**DOWN**	P09622;P09622-3;P09622-2	DLD	0.212202	18.5036	18.2914

Supplementary Table 5. Summary of protein quantification (LFQ) in RBC from DHST patients or HD controls, and in reticulocytes produced in vitro from CD34+ cells and exposed to DMSO or Yoda1.

Supplementary figure 1. Heatmap of log2 intensity (LFQ) of quantified proteins in at least 3 samples of at least one condition in purified RBC from DHST or HD individuals.

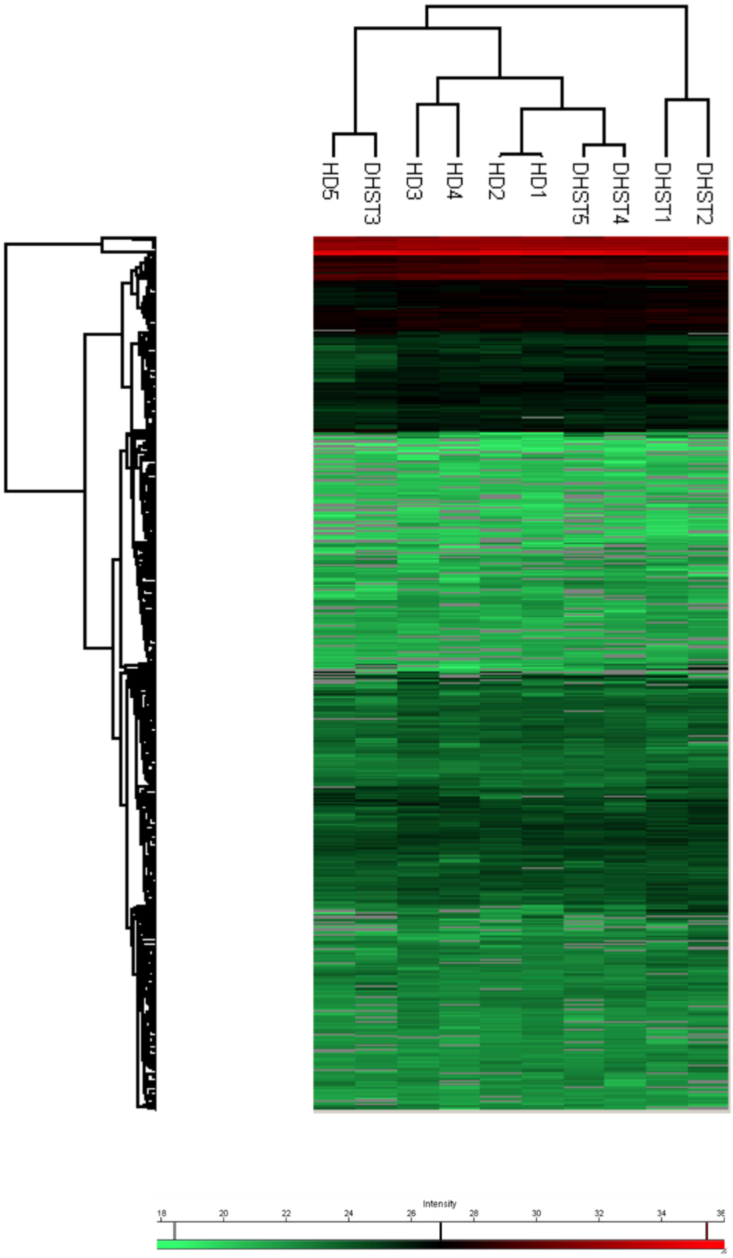

Supplementary figure 2. Histogram showing the absolute quantity of purified protein in each sample DHS *vs.* HD

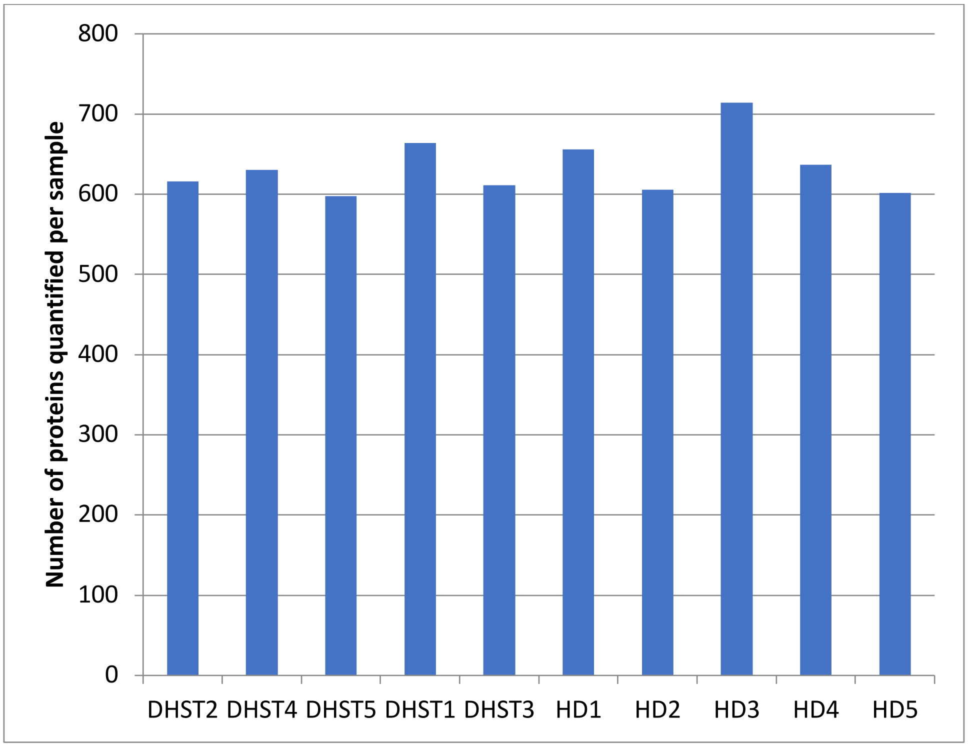

Supplementary figure 3. Heatmap of log2 intensity (LFQ) of quantified proteins in at least 3 samples of at least one condition in sorted reticulocytes from DMSO or Yoda1 samples.

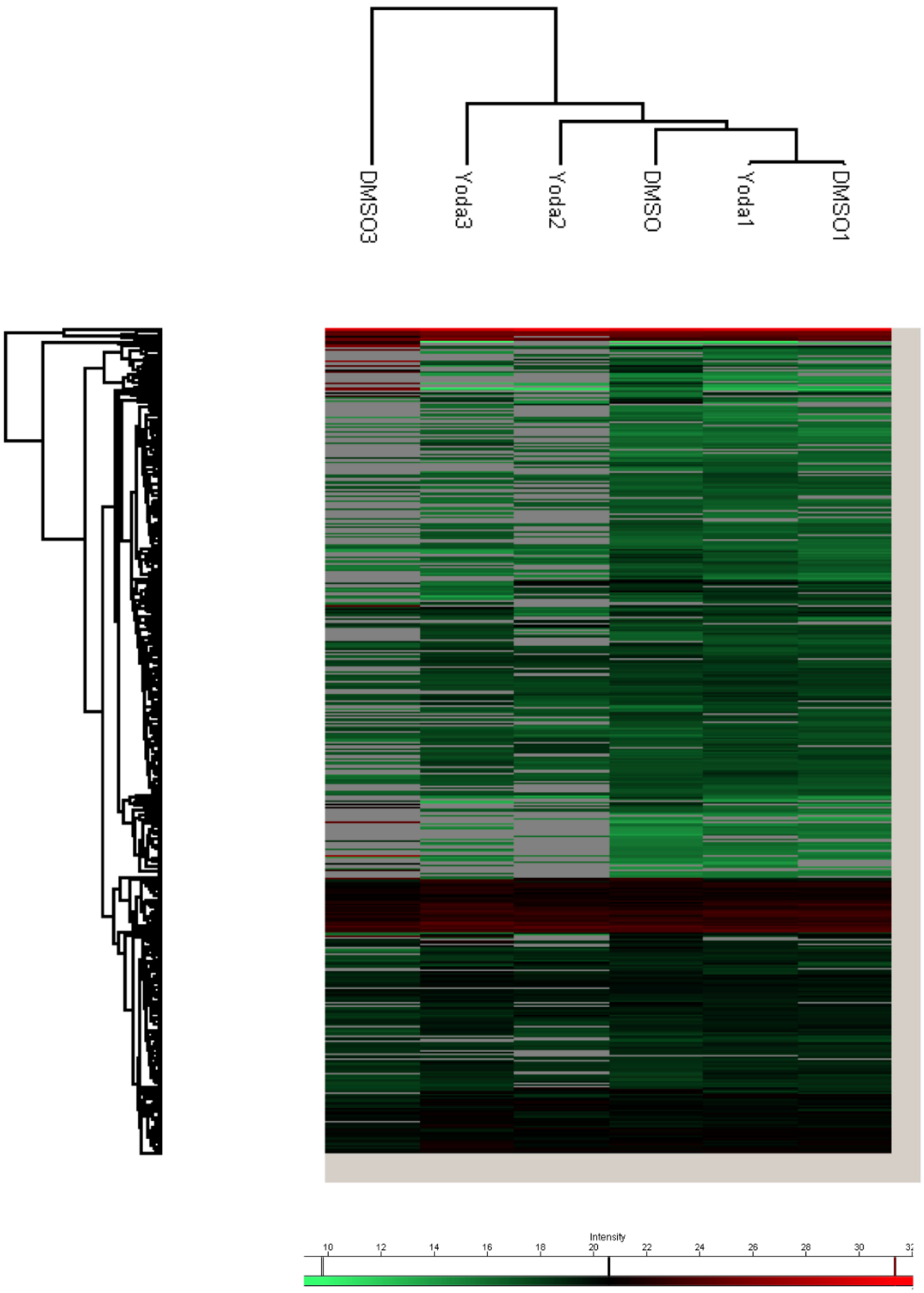

Supplementary figure 4: Histogram showing the absolute quantity of purified protein in each sample of sorted reticulocytes after exposure to DMSO *vs.* Yoda1

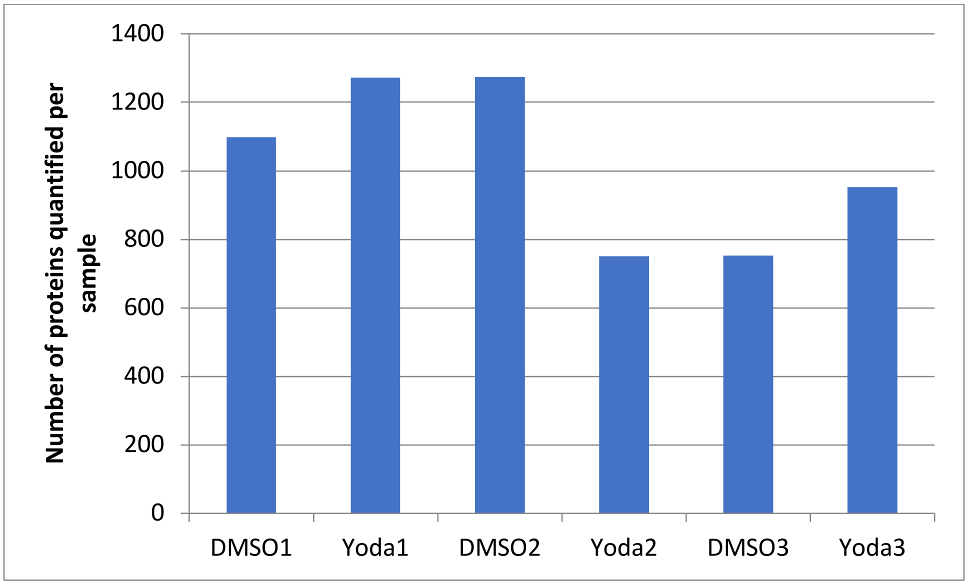

REFERENCES

Cox, J., Hein, M.Y., Luber, C.A., Paron, I., Nagaraj, N., Mann, M., 2014. Accurate proteome-wide label-free quantification by delayed normalization and maximal peptide ratio extraction, termed MaxLFQ. Mol Cell Proteomics 13, 2513–2526. https://doi.org/10.1074/mcp.M113.031591

Cox, J., Mann, M., 2008. MaxQuant enables high peptide identification rates, individualized p.p.b.-range mass accuracies and proteome-wide protein quantification. Nat Biotechnol 26, 1367–1372. https://doi.org/10.1038/nbt.1511

Kulak, N.A., Pichler, G., Paron, I., Nagaraj, N., Mann, M., 2014. Minimal, encapsulated proteomic-sample processing applied to copy-number estimation in eukaryotic cells. Nat Methods 11, 319–324. https://doi.org/10.1038/nmeth.2834

Picard, V., Guitton, C., Thuret, I., Rose, C., Bendelac, L., Ghazal, K., Aguilar-Martinez, P., Badens, C., Barro, C., Bénéteau, C., Berger, C., Cathébras, P., Deconinck, E., Delaunay, J., Durand, J.-M., Firah, N., Galactéros, F., Godeau, B., Jaïs, X., de Jaureguiberry, J.-P., Le Stradic, C., Lifermann, F., Maffre, R., Morin, G., Perrin, J., Proulle, V., Ruivard, M., Toutain, F., Lahary, A., Garçon, L., 2019. Clinical and biological features in *PIEZO1* -hereditary xerocytosis and Gardos channelopathy: a retrospective series of 126 patients. Haematologica 104, 1554–1564. https://doi.org/10.3324/haematol.2018.205328
