# Supplementary material for: Red blood cell proteomics reveal remnant protein biosynthesis and folding pathways in PIEZO1-related hereditary xerocytosis
Source: Front Physiol. 2022 Dec 1;13:960291. doi: 10.3389/fphys.2022.960291 (PMC9751340; doi:10.3389/fphys.2022.960291)
Supplement: Supplementary file 2 [file DataSheet1.docx]

**Supplementary Methods**

**Quantitative comparative global Label free proteomic approach:**

The proteomic approach used in the study is the quantitative label free approach (published by Cox et al., 2014). This approach allows relative and absolute quantification. Details of the analysis is described below.

**Process to obtain purified erythrocytes:**

Blood from HX patients and controls was centrifuged 10 minutes 150g. Plasma was isolated and centrifuged to eliminate platelets. Plasma depleted from platelets was then pooled with the red cell fraction. Cells were then separated using two different histopaque layers using Histopaque-1077 and Histopaque-1119 (Sigma-Aldrich) to remove mononuclear blood cells and granulocytes. To eliminate reticulocytes and obtain highly purified erythrocytes, nucleic acids were labelled with thiazole orange and erythrocytes were negatively sorted by FACS using a BD FACSJazz cell sorter.

**Sample processing for nLC-MS/MS analysis of erythrocytes:**

Cells were lyzed in lysis buffer (Tris HCl 200 mM, pH8.5, 2% SDS) and boiled 5 minutes at 95°C. Protein concentrations were determined using a bicinchoninic acid assay (BCA kit, Pierce). After reduction and alkylation in 20 mM TCEP [(tris(2-carboxyethyl)phosphine] and 50 mM Chloroacetamide, 50 µg of proteins were digested using FASP method. Eluted peptides were then fractionated using strong cation exchange (SCX) StageTips in 5 fractions as previously described (Kulak et al., 2014) and vacuum-dried while centrifuge in a Speed Vac (Eppendorf). Mass spectrometry (MS) analyses were performed on a Dionex U3000 RSLC nano-LC system coupled to an Orbitrap velos mass spectrometer (Thermo Fisher Scientific). Peptides from each SCX fraction were solubilized in 0.1% trifluoracetic acid containing 10% acetonitrile and separated on a C18 column (2-mm particle size, 75-mm inner diameter, 25-cm length; Thermo Fisher Scientific) with a 3-hour gradient starting from 99% solvent A (0.1% formic acid) and ending with 55% solvent B (80% acetonitrile, 0.085% formic acid). The mass spectrometers acquired data throughout the elution process and operated in a data-dependent scheme with full MS scans acquired with the LTQ-Orbitrap, followed by up to 20 MS/MS CID spectra in the Velos linear trap on the most abundant ions detected. Settings for Orbitrap Velos were: full MS automatic gain control (AGC): 1.10 ^6^ , maximum ion injection time (MIIT): 500 ms, resolution: 6.10^4^ , m/z range 400-2000 and for MS/MS, AGC: 5.10 ^3^, MIIT: 200 ms, minimum signal threshold: 500, isolation width: 2 Th, peptides with undefined charge state or charge state of 1 were excluded from fragmentation. Dynamic exclusion time was set to 30 s and spectra were recorded in profile mode.

**Data processing of erythrocyte data:**

The mass spectrometry data were analyzed using Maxquant version 1.5.3.30(Cox et al., 2014; Cox and Mann, 2008). The database used was a concatenation of human sequences from the Uniprot-Swissprot database (release 2017-05) and the list of contaminant sequences adapted from Maxquant. The enzyme specificity was trypsin. Carbamidomethylation of cysteins was set as constant modification and acetylation of protein N-terminus and oxidation of methionines were set as variable modification. Second peptide search was allowed and minimal length of peptides was set at 7 amino acids. False discovery rate (FDR) was kept below 1% on both peptides and proteins. Label-free protein quantification (LFQ) was done using both unique and razor peptides. At least 2 such peptides were required for LFQ ratio. The “match between runs” (MBR) option was allowed with a match time window of 1 min and an alignment time window of 30 min.

**Process to obtain cultured purified reticulocytes:**

CD34^+^ cells obtained from mobilized PBMCs of healthy donors were magnetically sorted on AutoMACS Separator (Miltenyi Biotec) using CD34^+^ magnetic antibodies (Miltenyi Biotec), after 30min incubation. CD34+ cells were then washed and cultured in erythroid differentiating media as described in Caulier et al., 2020. Yoda 1µM (Sigma-Aldrich) or iso-volume of DMSO was added to the media from day 1 to the end of the culture and refreshed at each media renewal time-point. At day 24, enucleated reticulocytes were co-stained using anti-human CD235a antibody (Miltenyi Biotec) and DRAQ5 cell-permeant nucleic acid staining (Thermo Fisher Scientifics) following manufacturer’s recommendation, then sorted by FACS using a BD FACSAriaII sorter.

**Sample processing protocol for reticulocytes:**

Cells were lyzed in lysis buffer (Tris HCl 200 mM, pH8.5, 2% SDS) and boiled 5 minutes at 95°C. Protein concentrations were determined using a bicinchoninic acid assay (BCA kit, Pierce). After reduction and alkylation in 20 mM TCEP [(tris(2-carboxyethyl)phosphine] and 50 mM Chloroacetamide, 50 µg of proteins were digested using FASP method. Eluted peptides were then fractionated using strong cation exchange (SCX) StageTips in 5 fractions as previously described (Kulak NA et al., Nat Methods, 2014) and vacuum-dried while centrifuge in a Speed Vac (Eppendorf). Mass spectrometry (MS) analyses were performed on a Dionex U3000 RSLC nano-LC system coupled to a Fusion mass spectrometer (Thermo Fisher Scientific). Peptides from each SCX fraction were solubilized in 0.1% trifluoracetic acid containing 10% acetonitrile and separated on a C18 column (2-mm particle size, 75-mm inner diameter, 25-cm length; Thermo Fisher Scientific) with a 3-hour gradient starting from 99% solvent A (0.1% formic acid) and ending with 55% solvent B (80% acetonitrile, 0.085% formic acid). MS data acquisition was performed throughout the elution process in a data-dependent scheme (top speed mode in 3 seconds) with full MS scans acquired with the orbitrap detector, followed by HCD peptide fragmentation and Ion trap fragment detection of the most abundant ions detected in the MS scan. Mass spectrometer settings for full scan MS were: 1.0E6 AGC, 60,000 target resolution, 350-1500 m/z range, maximum ion injection time (MIIT) of 60 ms. HCD MS/MS fragmentation was permitted for 2-7+ precursor ions reaching more than 5.0E3 minimum intensity. Quadrupole-filtered precursors within 1.6 m/z isolation window were fragmented with a Normalised Collision Energy setting at 30. 1.0E5 AGC Target and 60 ms MIIT were the limiting ions accumulation values. A 30 seconds dynamic exclusion time was set.

**Data processing protocol for reticulocyte data:**

The mass spectrometry data were analyzed using Maxquant version 2.0.3.0 (Cox et al., 2014; Cox and Mann, 2008). The database used was a concatenation of human sequences from the Uniprot-Swissprot database (release 2021-07) and the list of contaminant sequences adapted from Maxquant. The enzyme specificity was trypsin. Carbamidomethylation of cysteins was set as constante modification and acetylation of protein N-terminus and oxidation of methionines were set as variable modification. Second peptide search was allowed and minimal length of peptides was set at 7 amino acids. False discovery rate (FDR) was kept below 1% on both peptides and proteins. Label-free protein quantification (LFQ) was done using both unique and razor peptides. At least 2 such peptides were required for LFQ ratio. The “match between runs” (MBR) option was allowed with a match time window of 1 min and an alignment time window of 30 min.

**Supplementary Tables and Figures**

**Supplemental Tables 1 and 2**

Biological, genetics (Table 1) and clinical (Table 2) characteristics of the 5 PIEZO-1HX patients. (Picard et al., 2019). When multiple mutations are found in PIEZO1 sequence, the more likely to pathogenic is highlighted in black.

|  | **PIEZO1 mutation** | | **GR**  **(T/L)** | **Hb**  **(g/L)** | **VGM**  **(fL)** | **Retic**  **(G/L)** | **MCHC**  **(g/L)** | **MHC**  **(pg)** | **Proteins/cell (µg)** |
| --- | --- | --- | --- | --- | --- | --- | --- | --- | --- |
| **DHST1** | c.7479_7484dup | p.Glu2495_Leu2496dup | 2.81 | 102 | 100 | 221 | 362 | 36.3 | 60 |
| **DHST2** | c.1792G>C | p.Val598Leu | 3.61 | 132 | 103 | 320 |  | 34.2 | 59 |
| **DHST3** | c.6058G>A | p.Ala2020Thr | 4.6 | 140 | 83.2 | 161 | 363 | 30.2 | 42.15 |
| **DHST4** | c.2344G>A c.2423G>A  c.1792G>A | p.Gly782Ser  p.Arg808Gln  p.Val598Met | 4.2 | 147 | 99.6 | 225 | 350 | 34.9 | 66 |
| **DHST5** | c.2344G>A c.2423G>A  c.1792G>A | p.Gly782Ser  p.Arg808Gln  p.Val598Met | 2.5 | 171 | 92.7 | 260 | 355 | 32.9 | 52 |
| **HD1** |  |  | 5.07 | 150 | 88 |  | 334 | 29.5 | 49 |
| **HD2** |  |  |  |  |  |  |  |  |  |
| **HD3** |  |  | 4.72 | 134 | 85 |  | 336 | 28.4 | 48.8 |
| **HD4** |  |  | 4.99 | 141 | 85 |  | 239 | 28.2 | 48 |
| **HD5** |  |  |  |  |  |  |  |  |  |

|  | **Age/sexe** | **Hemolysis** | **Iron overload** | **Perinatal edema** | **Pseudohyperkaliema** |
| --- | --- | --- | --- | --- | --- |
| **DHST1** | 30/F | Y | N | Y | N |
| **DHST2** | 47/F | Y | N | NA | N |
| **DHST3** | 38/M | Y | Y | Y | Y |
| **DHST4** | 38/F | Y | N | Y | Y |
| **DHST5** | 58/F | Y | Y | N | Y |

**Supplemental Table 3.** Differentially expressed proteins between RBCs from HX (DHST) or control (HD) patients, and their corresponding gene names.

| Expression | Majority protein IDs | Gene names | Log2 Ratio DHST vs HD | Mean Log2 DHST | Mean Log2 HD |
| --- | --- | --- | --- | --- | --- |
| **UP** | P13639 | EEF2 | 3.53039 | 23.6766 | 20.1462 |
| **UP** | Q5VTE0;P68104;Q05639 | EEF1A1P5;EEF1A1;EEF1A2 | 2.86212 | 22.6271 | 19.765 |
| **UP** | P05387 | RPLP2 | 2.70475 | 24.7741 | 22.0693 |
| **UP** | P49327 | FASN | 1.68271 | 23.4464 | 21.7637 |
| **UP** | P22234 | PAICS | 1.59141 | 26.7542 | 25.1628 |
| **UP** | P49588 | AARS | 1.47477 | 21.7918 | 20.317 |
| **UP** | P41091;Q2VIR3 | EIF2S3;EIF2S3L | 1.42386 | 23.2385 | 21.8146 |
| **UP** | P61026 | RAB10 | 1.37945 | 23.7263 | 22.3468 |
| **UP** | Q9NZD4 | AHSP | 1.29019 | 27.1183 | 25.8281 |
| **UP** | O00410 | IPO5 | 1.23228 | 24.8268 | 23.5945 |
| **UP** | P61081 | UBE2M | 1.19741 | 23.0832 | 21.8858 |
| **UP** | Q13404 | UBE2V1 | 1.17481 | 24.6161 | 23.4412 |
| **UP** | Q8TEB1 | DCAF11 | 1.15555 | 21.0564 | 19.9009 |
| **UP** | P40227 | CCT6A | 1.05545 | 26.8018 | 25.7464 |
| **UP** | P05198 | EIF2S1 | 1.0208 | 22.7815 | 21.7607 |
| **UP** | Q9BRF8 | CPPED1 | 1.00322 | 23.6268 | 22.6235 |
| **UP** | P28838 | LAP3 | 0.975095 | 21.1423 | 20.1672 |
| **UP** | P78371 | CCT2 | 0.93137 | 27.9793 | 27.0479 |
| **UP** | Q9BXD5 | NPL | 0.918223 | 21.4047 | 20.4864 |
| **UP** | P49368 | CCT3 | 0.908663 | 26.4288 | 25.5201 |
| **UP** | P38606 | ATP6V1A | 0.893164 | 23.3632 | 22.47 |
| **UP** | Q14974 | KPNB1 | 0.889114 | 25.2223 | 24.3332 |
| **UP** | P17987 | TCP1 | 0.863143 | 26.3884 | 25.5253 |
| **UP** | P50990 | CCT8 | 0.853585 | 27.1768 | 26.3232 |
| **UP** | P50991 | CCT4 | 0.805005 | 26.6121 | 25.8071 |
| **UP** | Q9NQW7 | XPNPEP1 | 0.757181 | 21.7242 | 20.967 |
| **UP** | O95376 | ARIH2 | 0.755634 | 21.2815 | 20.5258 |
| **UP** | Q9H4A3 | WNK1 | 0.706381 | 23.0174 | 22.311 |
| **UP** | Q7Z6Z7 | HUWE1 | 0.699574 | 23.3426 | 22.643 |
| **UP** | Q9C0C9 | UBE2O | 0.696561 | 25.4433 | 24.7467 |
| **UP** | Q15181 | PPA1 | 0.681112 | 24.6693 | 23.9882 |
| **UP** | P35813 | PPM1A | 0.659594 | 22.977 | 22.3174 |
| **UP** | Q9Y570 | PPME1 | 0.594861 | 22.9409 | 22.346 |
| **UP** | Q99832 | CCT7 | 0.569999 | 26.2551 | 25.6851 |
| **UP** | O60610 | DIAPH1 | 0.539076 | 22.5966 | 22.0576 |
| **UP** | Q9H479 | FN3K | 0.513196 | 24.1412 | 23.628 |
| **UP** | Q14C86 | GAPVD1 | 0.500277 | 23.7397 | 23.2394 |
| **UP** | Q9H0P0 | NT5C3A | 0.490962 | 21.7565 | 21.2656 |
| **UP** | Q13619 | CUL4A | 0.484958 | 20.993 | 20.5081 |
| **UP** | P51858 | HDGF | 0.402851 | 21.922 | 21.5191 |
| **DOWN** | P04040 | CAT | -0.364452 | 31.4532 | 31.8177 |
| **DOWN** | O14818 | PSMA7 | -0.384709 | 25.3047 | 25.6894 |
| **DOWN** | Q14254 | FLOT2 | -0.44942 | 25.9827 | 26.4321 |
| **DOWN** | P30043 | BLVRB | -0.503589 | 29.4556 | 29.9591 |
| **DOWN** | Q15435 | PPP1R7 | -0.566836 | 22.2903 | 22.8571 |
| **DOWN** | Q9Y230 | RUVBL2 | -0.627031 | 22.587 | 23.214 |
| **DOWN** | P25685 | DNAJB1 | -0.633029 | 21.4903 | 22.1233 |
| **DOWN** | Q8WXX5 | DNAJC9 | -0.727389 | 20.0992 | 20.8266 |
| **DOWN** | P09972 | ALDOC | -0.739787 | 24.2868 | 25.0266 |
| **DOWN** | P00813 | ADA | -0.747832 | 19.8155 | 20.5634 |
| **DOWN** | P07451 | CA3 | -0.793649 | 24.9773 | 25.771 |
| **DOWN** | P52888 | THOP1 | -0.819527 | 21.4351 | 22.2546 |
| **DOWN** | P61978 | HNRNPK | -0.91101 | 22.7315 | 23.6426 |
| **DOWN** | P60983 | GMFB | -1.21584 | 21.815 | 23.0309 |
| **DOWN** | Q15185 | PTGES3 | -1.45803 | 21.6497 | 23.1077 |
| **DOWN** | P30046;A6NHG4 | DDT;DDTL | -1.90962 | 23.2856 | 25.1952 |

**Supplemental Table 4.** Differentially expressed proteins between cultured erythrocytes from HX (DHST) or control (HD) patients, and their corresponding gene names.

| Expression | Majority protein IDs | Gene names | Log2 Ratio DMSO vs Yoda1 | Mean Log2 DMSO | Mean Log2 Yoda1 |
| --- | --- | --- | --- | --- | --- |
| **UP** | P46926;P46926-2 | GNPDA1 | -0.181714 | 17.1973 | 17.379 |
| **UP** | O00410;O00410-3;O00410-2 | IPO5 | -0.214439 | 18.5136 | 18.728 |
| **UP** | P54727;P54727-2 | RAD23B | -0.290618 | 19.9471 | 20.2377 |
| **UP** | P08237-3;P08237;P08237-2 | PFKM | -0.373775 | 17.3758 | 17.7495 |
| **UP** | O15067 | PFAS | -0.374416 | 17.3303 | 17.494 |
| **UP** | P54920 | NAPA | -0.497541 | 18.7686 | 19.2662 |
| **UP** | P49591 | SARS | -0.507633 | 18.3372 | 18.8449 |
| **UP** | O60664-4;O60664;O60664-3 | PLIN3 | -0.528048 | 17.185 | 17.713 |
| **UP** | Q9NPB8 | GPCPD1 | -0.545201 | 15.4597 | 16.0049 |
| **UP** | P30566;P30566-2 | ADSL | -0.581664 | 19.24 | 19.8216 |
| **UP** | P48735;P48735-2 | IDH2 | -0.721919 | 19.7475 | 20.4694 |
| **UP** | O60784;O60784-4;O60784-2;O60784-3 | TOM1 | -0.791096 | 14.9676 | 16.0709 |
| **UP** | Q9BTU6 | PI4K2A | -0.803885 | 16.4078 | 17.2117 |
| **UP** | P62913-2;P62913 | RPL11 | -0.829947 | 18.1515 | 18.9815 |
| **UP** | Q15363 | TMED2 | -0.879981 | 15.089 | 15.969 |
| **UP** | P26641;P26641-2 | EEF1G | -0.947262 | 19.4551 | 20.4024 |
| **UP** | Q99714;Q99714-2 | HSD17B10 | -1.00174 | 17.4466 | 18.4484 |
| **UP** | Q96NA2 | RILP | -1.14654 | 17.1807 | 18.3272 |
| **UP** | P29692-2;P29692;P29692-4 | EEF1D | -1.5352 | 18.5803 | 20.1155 |
| **DOWN** | Q9UEY8-2;Q9UEY8 | ADD3 | 1.36835 | 16.7308 | 15.7695 |
| **DOWN** | O60841 | EIF5B | 1.24286 | 18.0322 | 16.7894 |
| **DOWN** | Q16658 | FSCN1 | 0.746424 | 17.3295 | 16.5831 |
| **DOWN** | P62873;P62873-2 | GNB1 | 0.671961 | 18.4198 | 17.7479 |
| **DOWN** | P00367;P00367-3;P00367-2;P49448 | GLUD1;GLUD2 | 0.560844 | 17.0853 | 16.5245 |
| **DOWN** | O43681 | ASNA1 | 0.498727 | 19.3087 | 18.81 |
| **DOWN** | P19623 | SRM | 0.429128 | 16.8525 | 16.3819 |
| **DOWN** | Q5XPI4;Q5XPI4-2 | RNF123 | 0.361284 | 17.9386 | 17.5773 |
| **DOWN** | P09622;P09622-3;P09622-2 | DLD | 0.212202 | 18.5036 | 18.2914 |

**Supplementary Table 5.** Summary of protein quantification (LFQ) in RBC from DHST patients or HD controls, and in reticulocytes produced in vitro from CD34+ cells and exposed to DMSO or Yoda1. PROVIDED AS A SEPARATE excel file

**Supplementary figure 1**. Heatmap of log2 intensity (LFQ) of quantified proteins in at least 3 samples of at least one condition in purified RBC from DHST or HD individuals.


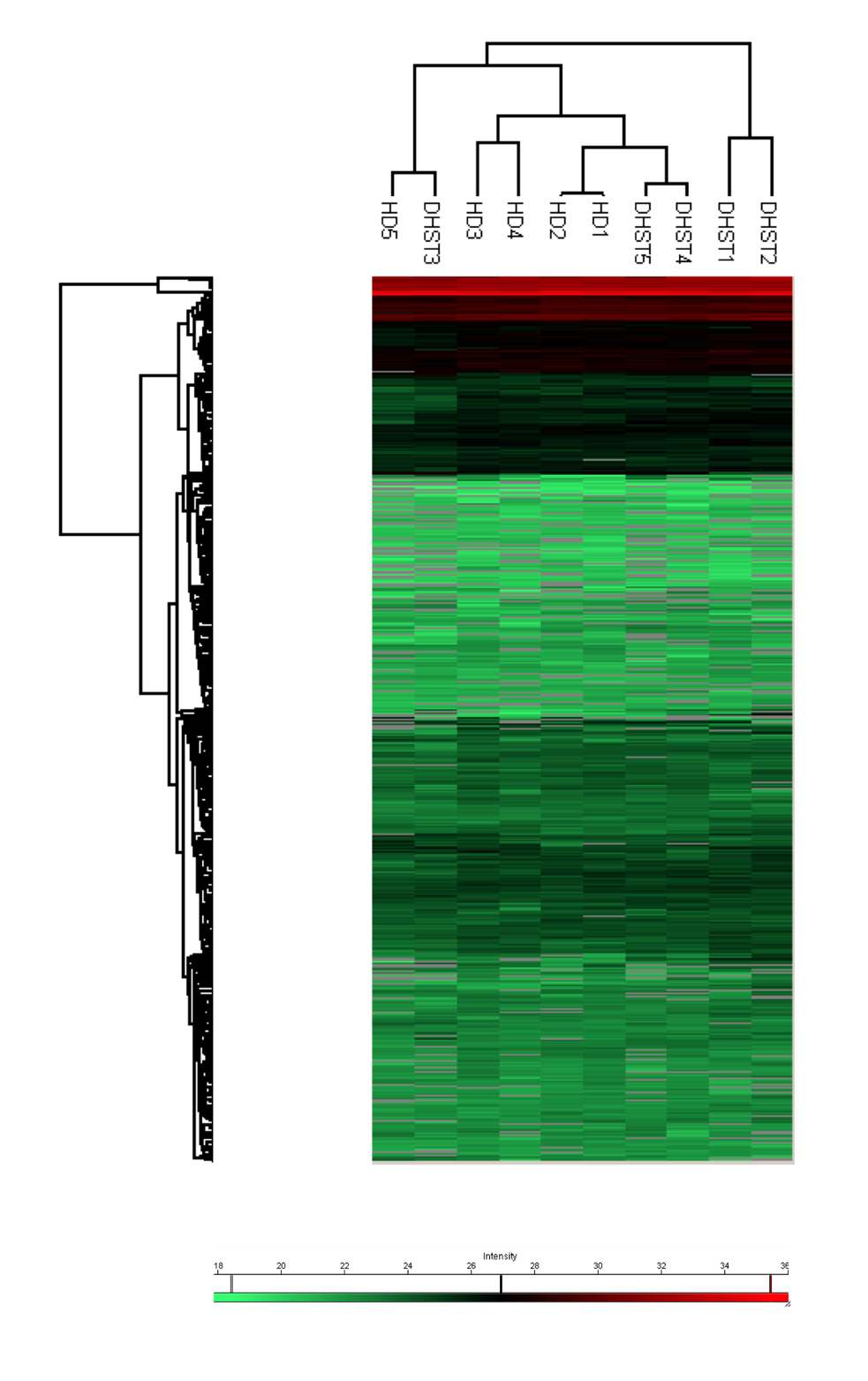


**Supplementary figure 2**. Histogram showing the absolute quantity of purified protein in each sample DHS *vs.* HD

**Supplementary figure 3.** Heatmap of log2 intensity (LFQ) of quantified proteins in at least 2 samples of at least one condition in sorted reticulocytes from DMSO or Yoda1 samples.


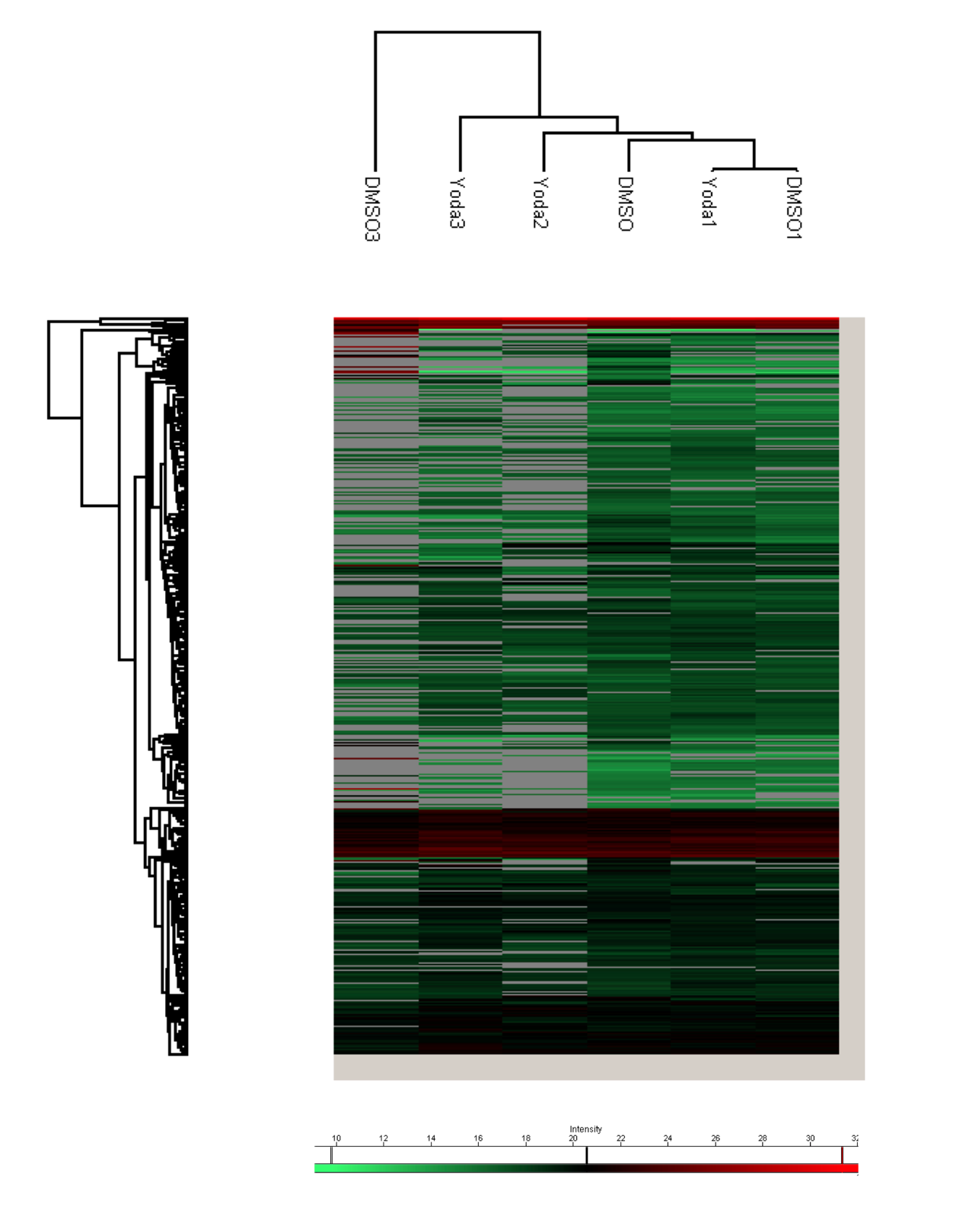


**Supplementary figure 4:**  Histogram showing the absolute quantity of purified protein in each sample of sorted reticulocytes after exposure to DMSO *vs.* Yoda1

**REFERENCES**

Cox, J., Hein, M.Y., Luber, C.A., Paron, I., Nagaraj, N., Mann, M., 2014. Accurate proteome-wide label-free quantification by delayed normalization and maximal peptide ratio extraction, termed MaxLFQ. Mol Cell Proteomics 13, 2513–2526. https://doi.org/10.1074/mcp.M113.031591

Cox, J., Mann, M., 2008. MaxQuant enables high peptide identification rates, individualized p.p.b.-range mass accuracies and proteome-wide protein quantification. Nat Biotechnol 26, 1367–1372. https://doi.org/10.1038/nbt.1511

Kulak, N.A., Pichler, G., Paron, I., Nagaraj, N., Mann, M., 2014. Minimal, encapsulated proteomic-sample processing applied to copy-number estimation in eukaryotic cells. Nat Methods 11, 319–324. https://doi.org/10.1038/nmeth.2834

Picard, V., Guitton, C., Thuret, I., Rose, C., Bendelac, L., Ghazal, K., Aguilar-Martinez, P., Badens, C., Barro, C., Bénéteau, C., Berger, C., Cathébras, P., Deconinck, E., Delaunay, J., Durand, J.-M., Firah, N., Galactéros, F., Godeau, B., Jaïs, X., de Jaureguiberry, J.-P., Le Stradic, C., Lifermann, F., Maffre, R., Morin, G., Perrin, J., Proulle, V., Ruivard, M., Toutain, F., Lahary, A., Garçon, L., 2019. Clinical and biological features in *PIEZO1* -hereditary xerocytosis and Gardos channelopathy: a retrospective series of 126 patients. Haematologica 104, 1554–1564. https://doi.org/10.3324/haematol.2018.205328
